# MicroRNA-139-5p upregulation is associated with diabetic endothelial cell dysfunction by targeting c-jun

**DOI:** 10.18632/aging.202257

**Published:** 2020-12-03

**Authors:** Yu-Fang Luo, Xin-Xing Wan, Li-Ling Zhao, Zi Guo, Rui-Ting Shen, Ping-Yu Zeng, Ling-Hao Wang, Jing-Jing Yuan, Wen-Jun Yang, Chun Yue, Zhao-Hui Mo

**Affiliations:** 1 Department of Endocrinology, Third Xiangya Hospital of Central South University and Diabetic Foot Research Center of Central South University, Changsha 410013, Hunan Province, China; 2 Key Laboratory of Hormones and Development (Ministry of Health), Tianjin Key Laboratory of Metabolic Diseases, Tianjin Metabolic Diseases Hospital and Tianjin Institute of Endocrinology, Tianjin Medical University, Tianjin 300070, China; 3 Center of Experimental Medicine, Third Xiangya Hospital of Central South University, Changsha 410013, Hunan Province, China

**Keywords:** endothelial colony forming cells, angiogenesis, diabetes, hind limb ischemia

## Abstract

Dysfunction of endothelial cells (ECs) and their progenitor cells is an important feature of diabetic vascular disease. MicroRNA (miR)-139-5p is involved in inhibiting the metastasis and progression of diverse malignancies. However, the role of miR-139-5p in ECs still remains unclarified. Here we demonstrated that miR-139-5p expression was elevated in endothelial colony-forming cells (ECFCs) isolated from patients with diabetes, ECs derived from the aorta of diabetic rodents, and human umbilical vein endothelial cells (HUVECs) cultured in high glucose media. MiR-139-5p mimics inhibited tube formation, migration, proliferation, and down-regulated expression of c-jun, vascular endothelial growth factor (VEGF), and platelet-derived growth factor (PDGF)-B, in ECFCs and HUVECs, respectively; moreover, miR-139-5p inhibitors reversed the tendency. Further, gain- and-loss function experiments and ChIP assay indicated that miR-139-5p regulate functions of ECFCs by targeting c-jun-VEGF/PDGF-B pathway. *In vivo* experiments (Matrigel plug assay and hindlimb ischemia model) showed that miR-139-5p downregulation further promoted ECFC-mediated angiogenesis and blood perfusion. In conclusion, diabetes-mediated high miR-139-5p expression inhibits the c-jun-VEGF/PDGF-B pathway, thus decreasing ECFCs migration, tube formation and proliferation, which subsequently reduces ECs survival. Therefore, miR-139-5p might be an important therapeutic target in the treatment of diabetic vasculopathy in the future.

## INTRODUCTION

Most diabetes-related deaths and disability are due to diabetic vascular complications, including lower extremity critical ischemic, the main cause of limb amputation [1]. In diabetic wound healing, attenuated angiogenic response is primary factor causing persistent foot ulcer [2]. Endothelial cells (ECs) dysfunction, one of the main features of diabetic vasculopathy, comprises impairment in migration, tube formation, proliferation and angiogenesis, as well as disturbances in vasodilation and vascular integrity [3]. Other hypotheses that delineate the mechanism of ECs dysfunction and subsequent impaired angiogenesis in diabetes include reduction in vascular endothelial growth factor-A (VEGF-A) signaling [4], accumulation of advanced glycation end products [5, 6], protein kinase C (PKC) activation [7], sorbitol-inositol imbalance [8], nuclear factor (NF)-κB-mediated downstream signaling [9], and the role of angiotensin-II and Peroxisome proliferator-activated receptor-gamma coactivator 1-α (PGC-1α) in the endothelium [10, 11]. Progenitor cells belonging to the endothelial lineage, have been shown to play critical roles in vascular integrity and postnatal vasculogenesis [12]. However, such endothelial progenitor cells (EPCs) from adult diabetics are reduced in number, which display blunted proangiogenic properties that impair vascular homeostasis and regeneration [13, 14].

MicroRNAs (miRNAs), a class of ~21 to 25 nucleotide long single-strand noncoding RNAs that typically suppress their target genes at the post-transcriptional or translational level, are involved in a variety of physiological and pathophysiological processes, including cancer and angiogenesis [15]. Among them, miR-139-5p has been found to inhibit metastasis and progression of multiple tumors [16]. Target genes of miR-139-5p that inhibit cell proliferation or migration and invasion include activator protein-1 (AP-1) [17], type 1 insulin-like growth factor receptor [18], endogenous autocrine motility factor receptor, NOTCH1 [19], and Rho-kinase 2 [20], most of which have been demonstrated to be involved in angiogenesis. Hence, we hypothesized that miR-139-5p has the potential to inhibit angiogenesis. Although the impact of miR-139 on pancreatic cancer ECs migration and angiogenesis has been demonstrated [21], the functional role and mechanistic action of miR-139-5p in a diabetic context remain unclear. Recently, Li et al. [22] reported that the expression of miR-139-5p in the pancreas of diabetic rats was increased and could be inhibited with liraglutide treatment, subsequently resulting in anti-apoptosis of islet cells. Besides, we found that the expression of miR-139-5p in mesenchymal stem cells (MSCs) from diabetic patients was up-regulated through microarray analysis (unpublished data). Thus, whether miR-139-5p has a potential role in mediating diabetic vascular complications by regulating the function of ECs has attracted our attention.

C-jun is a major component of the AP-1 transcription factor that promotes proliferation of breast and liver cancer [23, 24]. However, it is also required to counteract apoptosis [25]. Research has shown that the c-jun signaling pathway is integral to the proliferation and survival of pulmonary arterial ECs in case of pulmonary arterial hypertension-mediated cell damage [26]. Moreover, the c-jun pathway activates expression of the anti-apoptotic protein Bcl-XL in response to growth factor signaling, all of which protect primary human ECs from apoptosis [27].

Although vast evidence confirms the defective angiogenesis and vascular dysfunction in diabetes, its exact mechanism is unknown. Moreover, the role of miR-139-5p and c-jun (as predicted miR-139-5p’s biological target by TargetScan) in ECs dysfunction is unclear. Therefore, this study sought to find mechanistic evidence of miR-139-5p and c-jun in the regulation of endothelial colony-forming cells (ECFCs) in a diabetes-induced vascular dysfunction context.

## RESULTS

### Diabetes induces upregulation of miR-139-5p expression in endothelial cells

**Figure 1:**
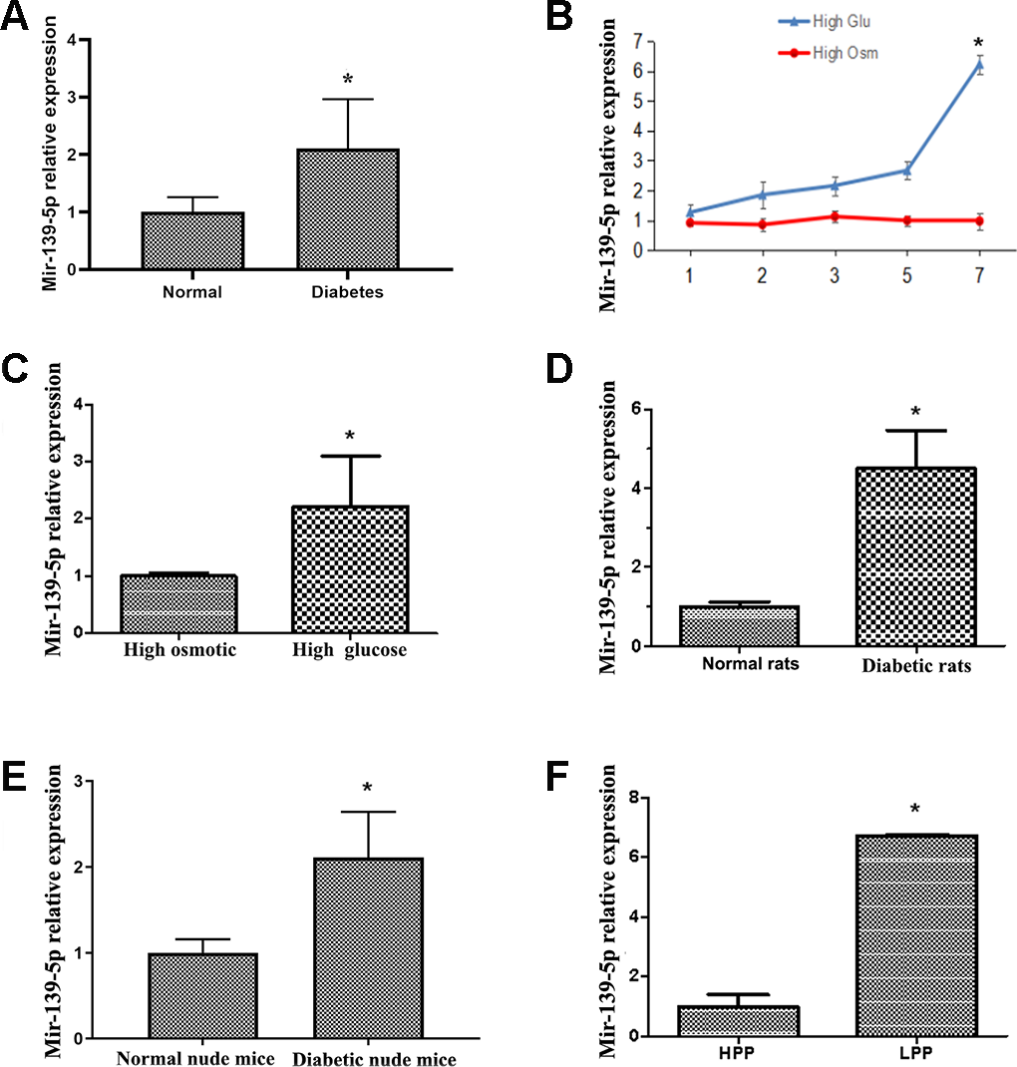
**Diabetes induces upregulation of miR-139-5p expression in endothelial cells.** (**A**) MiR-139-5p expression in endothelial colony-forming cells (ECFCs) isolated and cultured from peripheral blood (PB) of diabetic patients and healthy adults was detected by real-time PCR. (N=4) *P < .05 (**B**) MiR-139-5p expression in human umbilical vein endothelial cells (HUVECs) after 7 days of high glucose or high osmotic culture was detected by real-time PCR. (N=3) *P < .05 versus day 1 (**C**) MiR-139-5p expression in ECFCs from health PB after 7 days of high glucose or high osmotic culture was detected by real-time PCR. (N=3) *P < .05 versus day 1 (**D**) MiR-139-5p expression in endothelial cells derived from the thoracic aorta of normal and diabetic rats was detected by real-time PCR. (N=6) *P < .05 versus normal rats. (**E**) MiR-139-5p expression in endothelial cells derived from the thoracic aorta of normal and diabetic nude mouse was detected by real-time PCR. (N=6) *P < .05 versus normal nude mouse. (**F**) The expression of miR-139-5p in different clone cells. (N=3) *P < .05 versus high proliferation potential ECFCs. Data shown in the graphs represent mean ± standard deviation.

ECFCs were isolated and cultured from peripheral blood (PB) of patients with diabetes and healthy adults to test the effects of miR-139-5p on ECFCs in diabetes. Using real-time PCR, we found miR-139-5p expression in diabetic ECFCs to be 2-fold more than that of healthy subjects. MiR-139-5p expression was also high in ECFCs of healthy PB cultured in high glucose for 7 days *in vitro* compared with control groups cultured in normal glucose (Figure 1A, 1C). MiR-139-5p expression was 6-fold higher in high glucose media-cultured HUVECs than in high osmotic media-cultured HUVECs after 7 days. This induction was not associated with osmotic effects (Figure 1B). MiR-139-5p expression was similarly increased in ECs derived from STZ-induced diabetic rats and diabetic nude mice compared with normal rats and nude mice (Figure 1D, 1E). In addition, we found miR-139-5p expression to be significantly higher in the low proliferative potential ECFCs (LPP-ECFCs) than in the high proliferation potential ECFCs (HPP-ECFCs) isolated from healthy subjects (see description below, Figure 1F).

### MiR-139-5p impairs the angiogenic function of ECFCs and HUVECs *in vitro*

**Figure 2:**
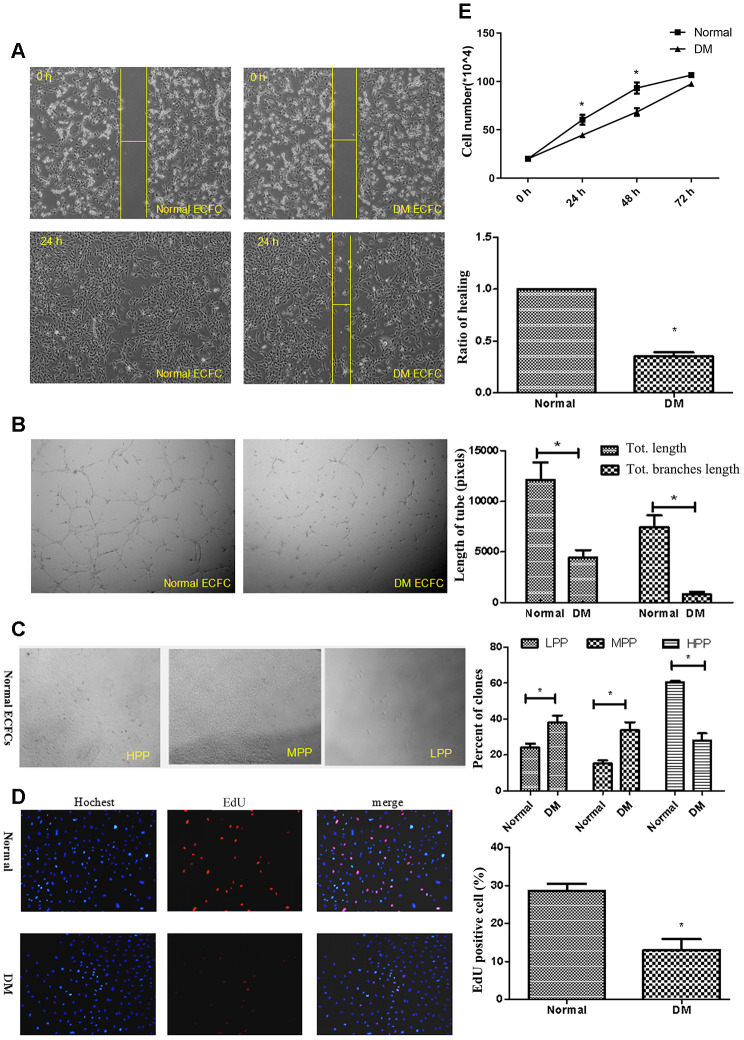
**ECFCs isolated from diabetic patients showed dysfunction.** (**A**) Scratch experiments detected the migration ability of ECFCs. (N=3) *P < .05 compared with normal ECFCs. (**B**) Matrigel assays performed for tube formation experiments identified the angiogenesis of ECFCs. The photos were captured with a 40X microscope. (N=3) *P < .05 compared with normal ECFCs. (**C**) Single cloned cell proliferation experiments detected the proliferation ability of single cells. Cell number <500 was low clone (LPP), <10,000 was normal clone (MPP), and >10,000 was high clone (HPP). The cell was seeded into a 96-well plate and the ratio of clones was counted. (N=3) *P < .05 versus normal ECFC. (**D**, **E**) The proliferative ability of diabetic ECFCs was found to be impaired. The nucleus was dyed blue by Hoechst stain and the proliferating cells were dyed red by 5-ethynyl-2′-deoxyuridine. The photos were captured by a 40X microscope. (N=3) (**D**). Hemocytometer counting of ECFCs in 24, 48, and 72h after seeding at the density of 2x10^4^cells/well. (N=3) *P < .05 versus normal ECFCs (**E**). Data shown in the graphs represent mean ± standard deviation.

ECFCs isolated from diabetic patients showed blunted migration in scratch assays and significantly reduced vessel forming in tube formation assay compared with cells from healthy subjects (Figure 2A, 2B). Using single cell clonogenic assays, we quantified the clonal formation and proliferation potential of HPP-ECFCs (HPP-ECFCs >10^4^ cells/colony), medium (500-10^4^ cells/colony), and LPP-ECFCs (LPP-ECFCs <500 cells/colony) as described previously [28]. Clonal formation percentages of high, medium, and low proliferative potential-ECFCs in healthy PB-derived ECFC were 60.5%, 15.8%, and 24.2%, respectively, whereas the percentages in diabetic patient PB-derived ECFCs were 28%, 34%, and 38%, respectively, after 14 days of culture. Clonal formation percentage of HPP-ECFCs in diabetic patients was significantly lower, whereas the proportion of medium and low proliferative potential-ECFCs was significantly higher than that of healthy adults (*P*<.05; Figure 2C). Cell proliferation in diabetic-derived ECFCs was significantly lower (*P*<.05 for all) than healthy subjects-derived ECFCs, as observed by Hoechst stain, 5-ethynyl-2′-deoxyuridine (EdU) dye cell, and cell counting experiments (Figure 2D, 2E).

**Figure 3:**
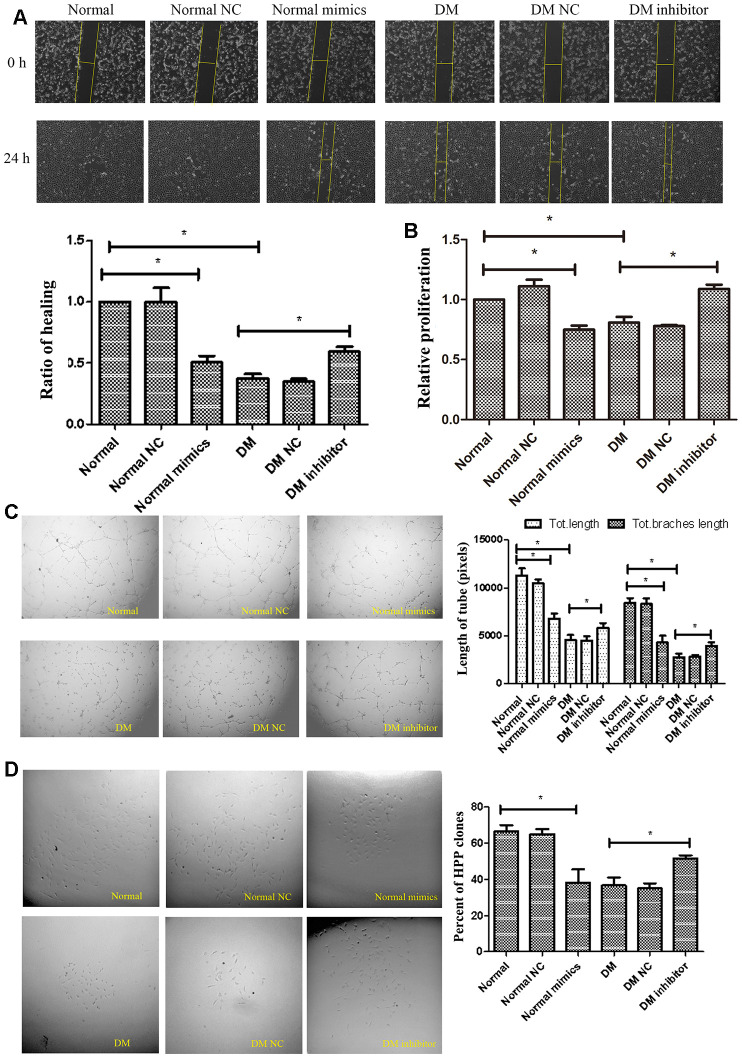
**MiR-139-5p inhibits the function of ECFCs.** (**A**, **B**) Migration and proliferation of normal ECFCs transfected with miR-139-5p mimics and DM ECFCs transfected with miR-139-5p inhibitors were assessed. The photos were captured by a 40X microscope and the statistics performed by Image J. (N=3) *P < .05 versus normal or diabetic ECFCs. (**C**) Tube formation of normal ECFCs transfected with miR-139-5p mimics and DM ECFCs transfected with miR-139-5p inhibitors were assessed. The photos were captured by a 40X microscope and the statistics performed by Image J. (N=3) *P < .05 versus normal or diabetic ECFCs. (**D**) The cloning ability of normal ECFCs transfected with miR-139-5p mimics and diabetic ECFCs transfected with miR-139-5p inhibitors were detected using single cell clone experiments. (N=3) *P < .05 versus normal or diabetic ECFCs. Data shown in the graphs represent mean ± standard deviation.

The impairment of ECFCs migration coinciding with induction of miR-139-5p expression suggested that miR-139-5p may inhibit ECFCs cloning and mediating endothelial cell dysfunction in diabetes mellitus. To confirm this hypothesis, ECFCs from healthy subjects were transfected with miR-139-5p mimics and inhibitors, respectively; the corresponding increase or decrease in miR-139-5p expression was observed (Supplementary Figure 1A, 1B). Tube formation and scratch assays showed that usage of the miR-139-5p mimics inhibited the formation of tube-like structures and migration in health-derived ECFCs, respectively; conversely, miR-139-5p inhibitors rescued the formation of tube-like structures and migration in diabetic-derived ECFCs, respectively. (Figure 3A, 3C). Similarly, miR-139-5p mimics (in normal ECFCs) significantly decreased the proliferative potential, while miR-139-5p inhibitors (in diabetic ECFCs) significantly reversed the tendency (*P*<.05 for all), as detected by the CCK8 assay and single cell cloning assays (Figure 3B, 3D). Moreover, HUVECs transfected with miR-139-5p mimics displayed significantly repressed formation of tube-like structures and migration ability, while transfected with miR-139-5p inhibitors displayed significantly enhanced formation of tube-like structures and migration ability, respectively (*P*<.05 for all; Supplementary Figure 2A, 3B). These data indicated that miR-139-5p is a negative regulator of the functions of ECFCs and HUVECs.

### MiR-139-5p targets c-jun to inhibit the function of ECFCs and HUVECs

**Figure 4:**
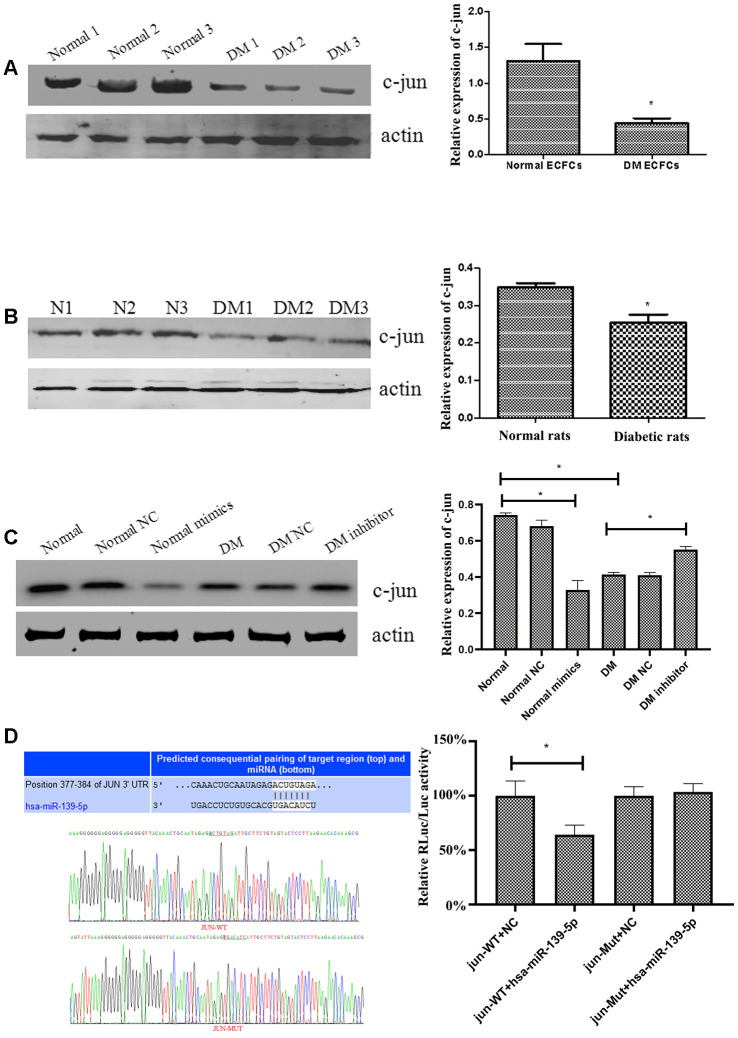
**MiR-139-5p regulates the expression of c-jun.** (**A**) C-jun expression was detected by Western blot in ECFCs derived from diabetic patients and healthy subjects. (N=3) *P < .05 versus normal ECFCs. (**B**) C-jun expression was detected by Western blot in ECs derived from the thoracic aorta of diabetic rats and normal rats. (N=3) *P < .05 versus normal rats. (**C**) C-jun expression was detected by Western blot in normal ECFCs transfected with a miR-139-5p mimic and in diabetic ECFCs transfected with a miR-139-5p inhibitor. (N=3) *P < .05 versus normal or diabetic ECFCs. (**D**) Dual Luciferase Reporter Assay used to assess whether c-jun was the target of miR-139-5p. (N=3) *P < .05. Data shown in the graphs represent mean ± standard deviation.

Although c-jun regulation by miR-139 has been reported previously in human gastric cancer cells [17], the same has not been studied in ECs and ECFCs. Western blot showed a significant decrease in c-jun expression (*P*<.05 for all) in ECFCs from diabetic patients compared with the health individuals (Figure 4A) and in ECs derived from diabetic rats compared with normal rats (Figure 4B). Furthermore, treating ECFCs with miR-139-5p mimics or inhibitors downregulated or upregulated c-jun expression (Figure 4C), respectively, indicating that c-jun may be a target gene of miR-139-5p.

As TargetScan predicted, the 3'-UTR of c-jun contains a conserved binding site in the ACTGTAG position for miR-139-5p (Figure 4D). We used a Dual Luciferase Reporter Assay to identify whether the c-jun 3'-UTR can be recognized by miR-139-5p and thus to confirm the prediction. The pmiR-RB-REPORT^TM^ vector was chosen to perform this detection in the assay. ECFCs were transiently co-transfected with miR-139-5p mimics and wild-type (Luc-c-jun-3'-UTR) or mutated (Luc-c-jun-mut3'-UTR) reporter plasmid (RLuc representing the report luciferase and Luc the control luciferase). The relative RLuc/Luc ratio of ECFCs co-transfected with miR-139-5p and wild-type-c-jun 3'-UTR decreased significantly (*P*<.05), whereas a significant change in the relative RLuc/Luc ratio of ECFCs co-transfected with miR-139-5p and Mut-c-jun 3'-UTR was not observed (Figure 4D). This revealed that miR-139-5p could directly target and decrease c-jun expression.

**Figure 5:**
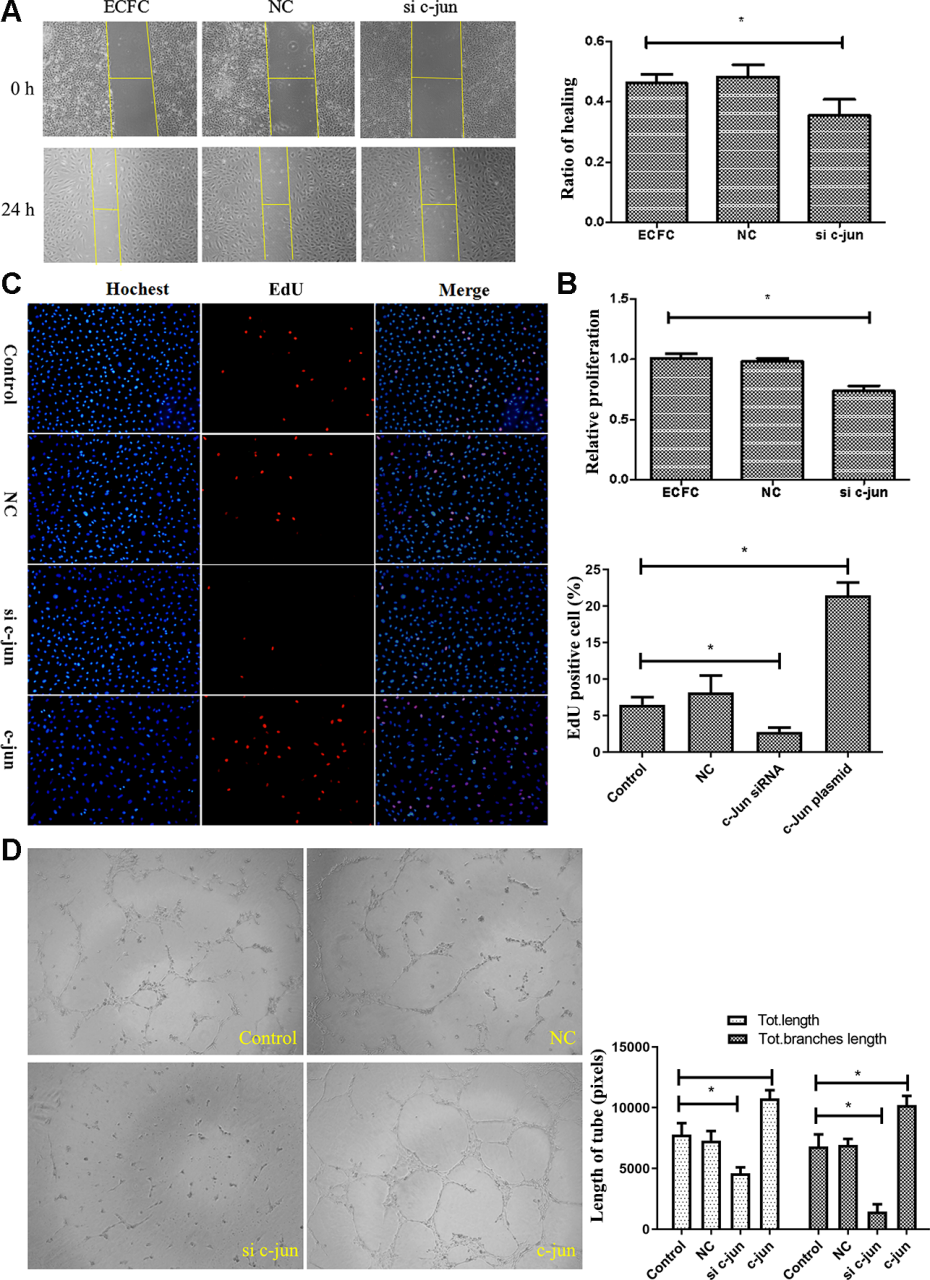
**Effect of c-jun on ECFCs.** (**A**) Scratch assay detected the migration ability of ECFC transfected with c-jun siRNA. (N=3). (**B**, **C**) CCK8 and cell counting detected the proliferative ability of ECFCs treated with c-jun siRNA or overexpress plasmid. The nucleus was dyed blue by Hoechst stain and the proliferating cells were dyed red by 5-ethynyl-2′-deoxyuridine. The photos were captured by a 40X fluorescence microscope. (N=3) *P < .05 versus control. (**D**) *In vitro* tube formation experiments detected the angiogenesis ability of ECFCs with different treatment. The photos were captured by a 40X fluorescence microscope. (N=3) *P < .05 versus control. Data shown in the graphs represent mean ± standard deviation.

To verify the regulation of c-jun in ECFCs function, the effects of c-jun overexpression or knockdown on ECFCs were studied. Transfected diabetic-derived ECFCs with c-jun siRNA showed reduced proliferation as estimated in the CCK8 assay and the Hoechst and EdU stain experiments (Figure 5B, 5C), repressed migration as detected by scratch assay (Figure 5A), and decreased vessel formation in tube formation assay (Figure 5D). On the other hand, overexpression of c-jun rescued ECFCs vessel formation and also improved the proliferative potential of ECFCs (*P*<.05for all). C-jun was thus found to be an important regulator of ECFCs angiogenesis and proliferation.

**Figure 6:**
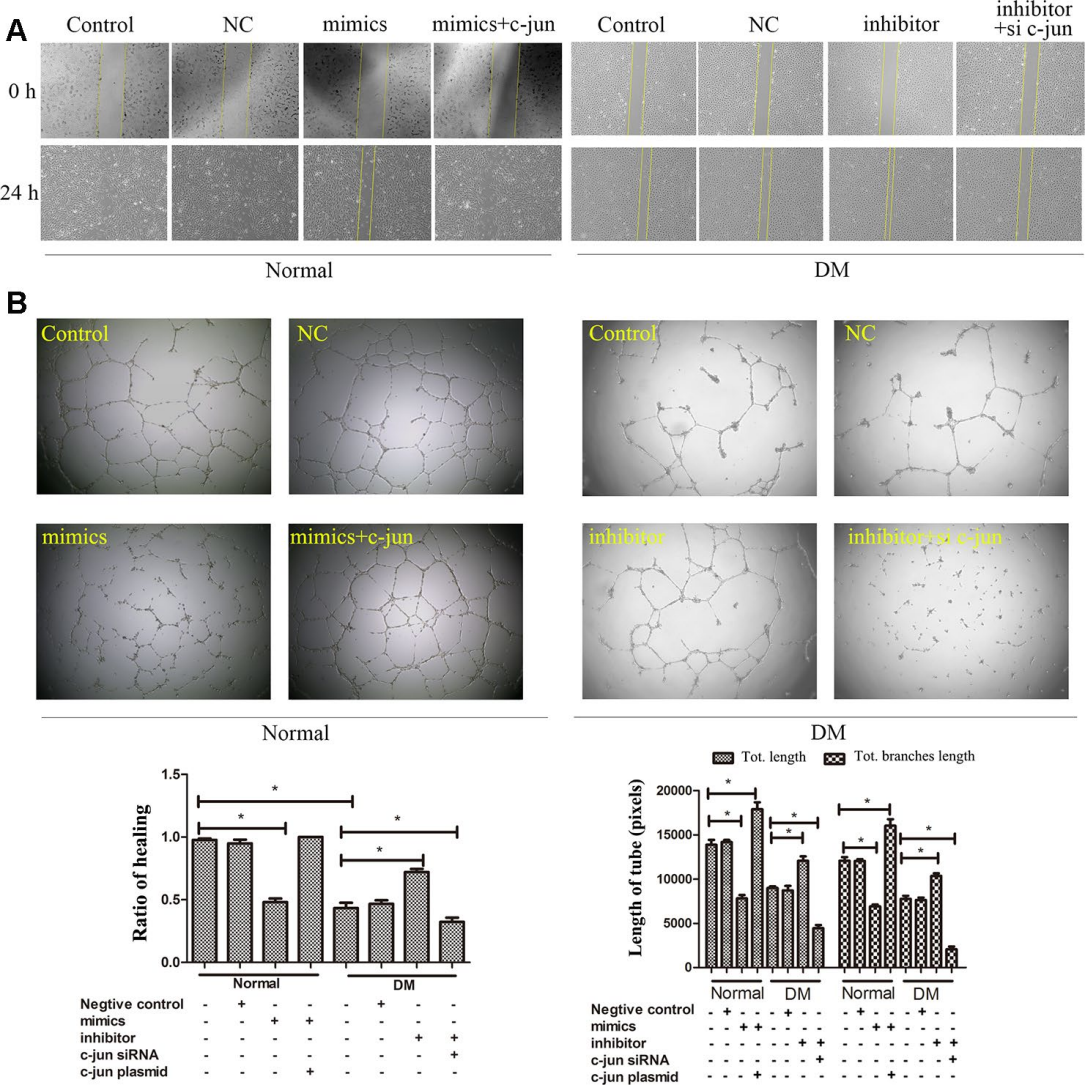
**MiR-139-5p-mediated c-jun expression regulates the function of ECFCs.** The migration (**A**) and angiogenesis (**B**) of normal ECFCs were co-transfected with miR-139-5p mimics and c-jun plasmids and diabetic ECFCs were co-transfected with miR-139-5p inhibitors and c-jun siRNA. The photos were captured by a 40X fluorescence microscope. (N=3) *P < .05 versus control. Data shown in the graphs represent mean ± standard deviation.

We further investigate whether miR-139-5p-mediated c-jun expression could regulate the function of ECFCs. Co-transfected health-derived ECFCs with c-jun plasmid could rescue the decreased migration and tube formation abilities induced by miR-139-5p mimics, respectively; however, co-transfected with si-c-jun could deprive the increased migration and tube formation capacities induced by miR-139-5p inhibitors in diabetic-derived ECFCs, respectively (Figure 6A, 6B). Thus, we concluded that miR-139-5p targets c-jun to inhibit the functions of ECFCs. Furthermore, western blot experiments found no change in IGF-1R, an angiogenesis-related protein, which also contains a conserved binding site for miR-139-5p (data not shown).

### MiR-139-5p inhibits VEGF and PDGF-B expressions by c-jun to impair the function of endothelial cells

**Figure 7:**
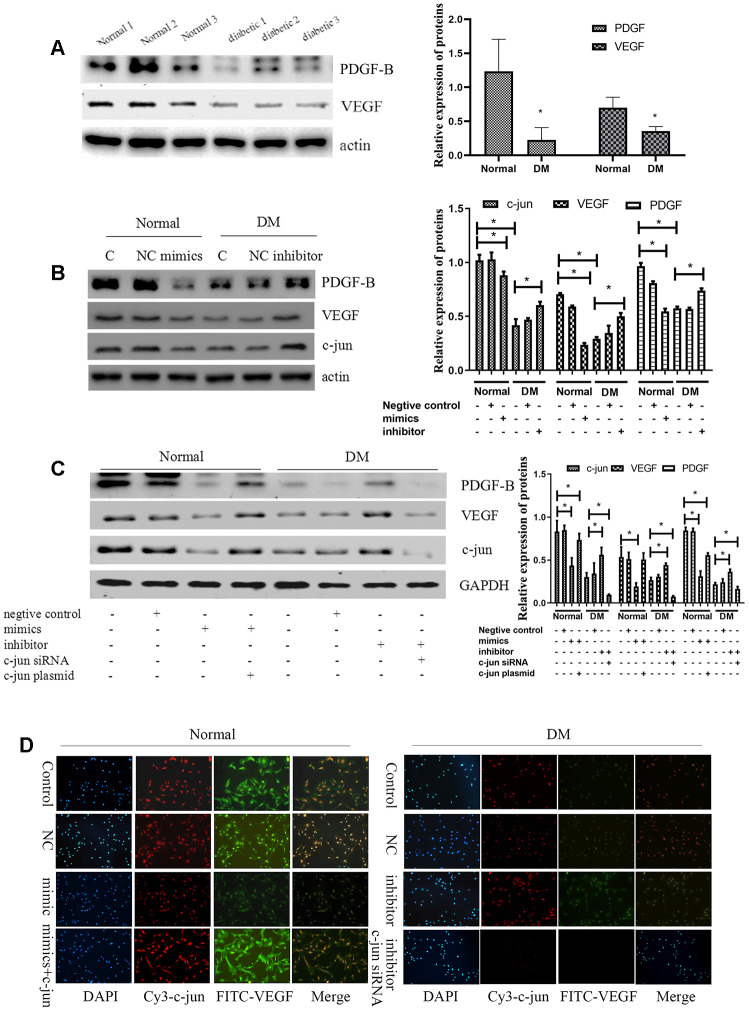
**MiR-139-5p inhibits VEGF expression by c-jun to inhibit the function of ECFCs.** (**A**) PDGF-B and VEGF expression detected by Western blot in healthy and diabetic subjects. (N=3) *P < .05 versus normal ECFCs. (**B**) C-jun, VEGF, and PDGF-B expression in normal ECFCs transfected with miR-139-5p mimics and diabetic ECFCs transfected with a miR-139-5p inhibitors. (N=3) *P < .05 versus normal or diabetic ECFCs. (**C**) C-jun, VEGF, and PDGF-B expression was detected when normal ECFCs were transfected with miR-139-5p mimics and c-jun plasmids and diabetic ECFCs were transfected with miR-139-5p inhibitors and c-jun siRNA. (N=3) *P < .05 versus normal or diabetic ECFCs. (**D**) C-jun and VEGF expression was detected in normal ECFCs co-transfected with miR-139-5p mimics and c-jun plasmid, whereas diabetic ECFCs were co-transfected with miR-139-5p inhibitors and c-jun siRNA. The immunofluorescence was used to identify the c-jun and VEGF expression. Red indicates Cy3-labeled c-jun and green indicate FITC-labeled VEGF. The photos were captured by a 40X fluorescence microscope. (N=3) *P < .05. Data shown in the graphs represent mean ± standard deviation.

VEGF and PDGF-B decreased significantly in diabetic-derived ECFCs as detected by Western blot and immunofluorescence (*P*<.05; Figure 7A, 7D). Next, we found that transfection with miR-139-5p mimics could decreased the expression of VEGF and PDGF-B in ECFCs and HUVECs, respectively; miR-139-5p inhibitors could reverse the tendency (*P*<.05 for all; Figure 7B and Supplementary Figure 2C). These results coincided with the induction of c-jun expression by miR-139-5p in ECFCs (Figure 7B). Western blot verified that overexpression of c-jun could elevated the expression of VEGF and PDGF-B, respectively; whereas downregulation of c-jun reversed the tendency (Supplementary Figure 3). Moreover, transfecting ECFCs with a c-jun plasmid rescued miR-139-5p mimic-induced decrease in VEGF and PDGF-B, whereas transfecting diabetic ECFCs with c-jun siRNA blocked miR-139-5p inhibitor-mediated PDGF-B and VEGF induction (*P*<.05 for all; Figure 7C, 7D). These results indicate that miR-139-5p inhibits VEGF and PDGF-B expression at least partly via c-jun.

**Figure 8:**
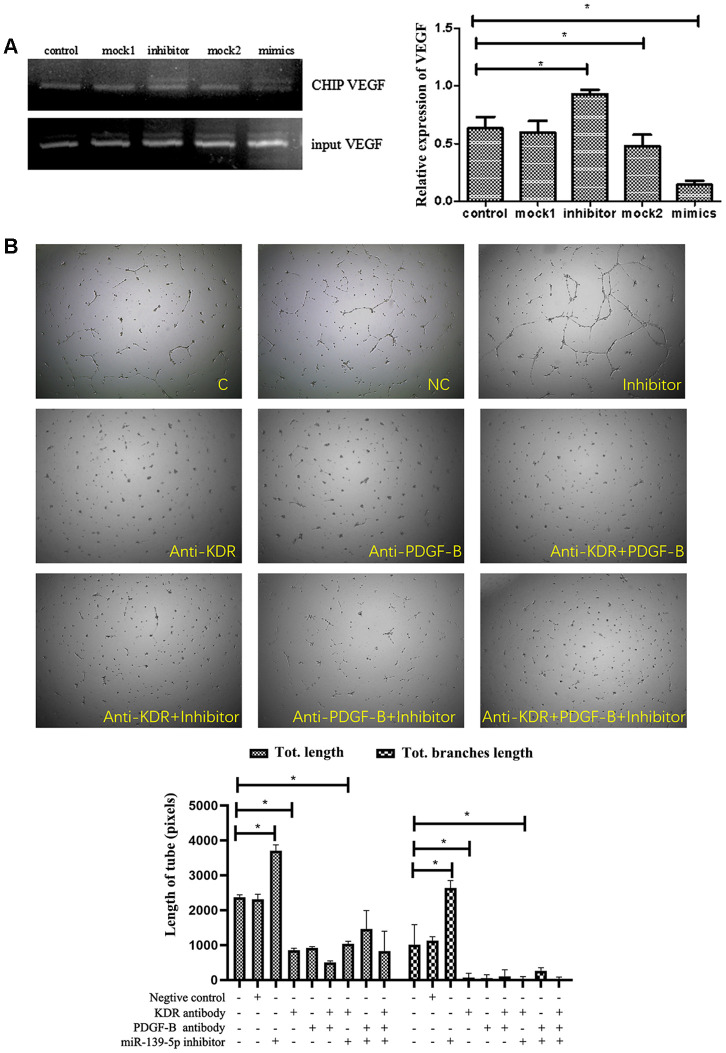
**The function of miR-139-5p inhibitors depends on PDGF-B and VEGF expression.** (**A**) The different treated ECFCs were lysed and used to perform ChIP experiments. C-jun antibody was used to bind the VEGF promoter while a special VEGF primer was used to detect the binding *vegf* or gDNA *vegf* in the ChIP or input DNA. The input VEGF was the PCR product of total DNA without immunoprecipitation + c-jun antibody, which was used as the normalization control. The ChIP VEGF was the PCR product of the DNA bound to the c-jun antibody. (N=3) *P < .05 versus control. (**B**) Tube formation of diabetic-derived ECFCs transfected with miR-139-5p inhibitors with or without anti-KDR/anti-PDGF treatment was observed. The photos were captured by a 40X microscope. (N=3) *P < .05. Data shown in the graphs represent mean ± standard deviation.

Researchers have suggested that c-jun/AP-1 could bind to the promoter region of VEGF and PDGF-B gene and thus increase VEGF and PDGF-B transcription [29, 30]. In the present research, we also tried to confirm miR-139-5p could inhibit VEGF expression by decreasing the activity of c-jun and VEGF promoter in ECFCs through ChIP experiments. MiR-139-5p mimic treatment significantly decreased c-jun-VEGF binding (*P*<.05), whereas input VEGF did not cause significant change compared with the control group (Figure 8A). These results indicate that miR-139-5p inhibited VEGF expression through reduction in c-jun-VEGF promoter activity. Afterward, we treated ECFCs with anti-kinase insert domain receptor (KDR, the VEGF tyrosine kinase cell receptors-2 in human) [31] and anti-PDGF-B, or both. The results showed that the formation of tube-like structures induced by miR-139-5p inhibitor in ECFCs was blocked (Figure 8B). Therefore, it is believed that VEGF and PDGF-B are involved in miR-139/c-jun-mediated endothelial cell angiogenesis.

### MiR-139-5p upregulation prevents tube formation of ECFCs and miR-139-5p downregulation promotes tube formation of ECFCs *in vivo*

**Figure 9:**
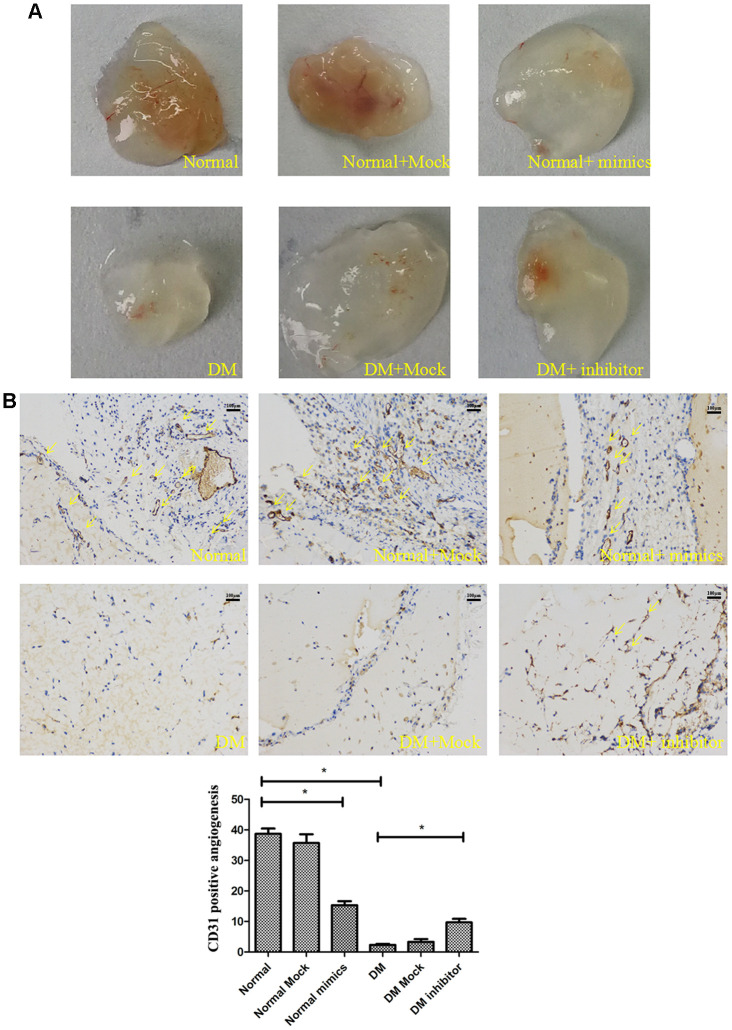
**MiR-139-5p prevents tube formation *in vivo*.** Matrigel was mixed with the normal ECFCs transfected with miR-139-5p mimics or diabetic ECFCs transfected with miR-139-5p inhibitors. The mixed Matrigel was inoculated into the abdomen of nude mice. After 2 weeks, the Matrigel was separated from euthanized mice. (**A**) Representative photographs. (N=4) *P < .05 versus normal or diabetic group. (**B**) CD31 histological images under high-power fields (200X) of normal optical microscope. (N=3) *P < .05 versus normal or diabetic group. Data shown in the graphs represent mean ± standard deviation.

Matrigel plug assays were performed *in vivo* to determine whether miR-139-5p regulates new blood vessel-forming ability of ECFCs. Health-derived ECFCs transfected with a miR-139-5p mimics or diabetic-derived ECFCs transfected with miR-139-5p inhibitors were mixed with Matrigel and injected subcutaneously into the abdomen of nude mice. Both representative photographs of the Matrigel (Figure 9A) and CD31 histological (Figure 9B) experiments showed the miR-139-5p mimics prevent tube formation of health-derived ECFCs and the miR-139-5p inhibitors promote tube formation of diabetic-derived ECFCs.

### Downregulation of miR-139-5p expression promotes ECFC-mediated angiogenesis and blood perfusion in diabetic HLI mice model

**Figure 10:**
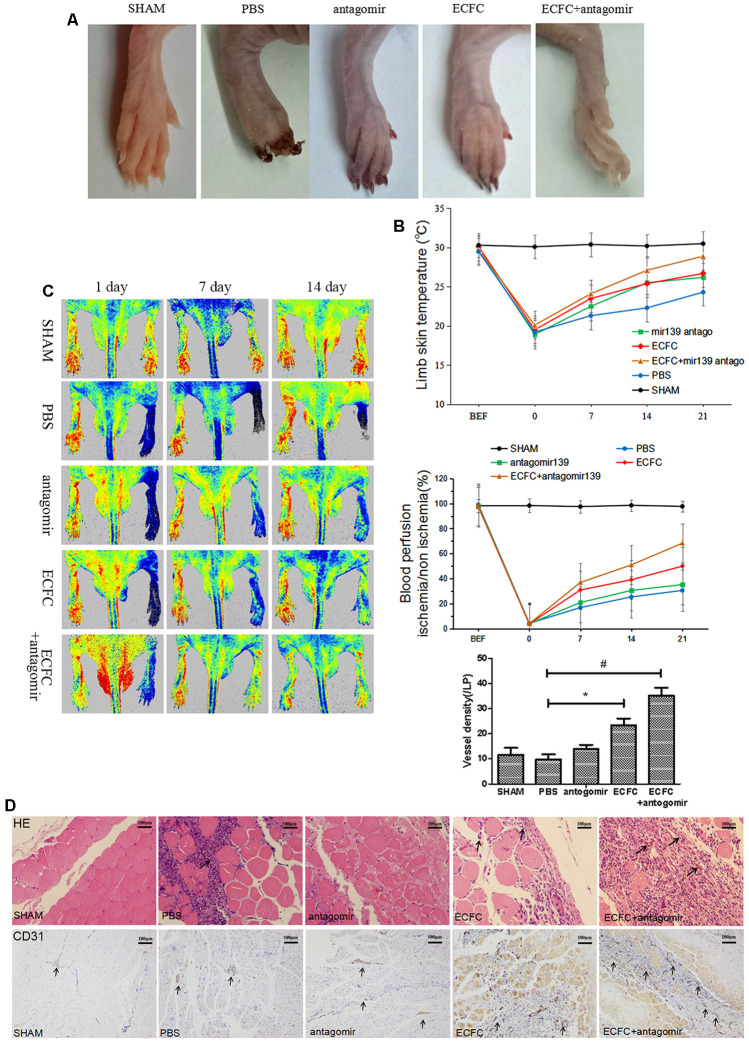
**Downregulating miR-139-5p expression promotes ECFC-mediated angiogenesis and blood perfusion in hind limb ischemia (HLI) in diabetic mice.** (**A**) Representative ischemia photographs of 5 groups taken 14 days after injections. Group A: Sham; Group B: PBS; Group C: antagomiR-139; Group D: ECFC; Group E: ECFCs + antagomiR-139. (**B**) Time-dependent effect of ECFC and/or antagomiR-139 treatment on skin temperature of lower extremity in ischemic mice. (N=3) *P<.05 and ^#^*P*<.01, both versus PBS. (**C**) Improved limb blood perfusion by transplanting ECFC and antagomir-139-5p in ischemic limb of diabetic mice. The time course of blood perfusion is shown in the images and quantitative analysis after HLI surgery with or without ECFC transplantation and/or antagomir-139-5p. Blood perfusion is the ratio of ischemic to non-ischemic limb perfusion measured by a PeriCam Perfusion Speckle Imager. (N=3) *P<.05 and ^#^*P*<.01, both versus PBS. (**D**) Effect of ECFC transplantation and/or antagomiR-139 on vascular density of lower extremity in ischemic mice. Vascular density was determined by counting the number of CD31-stained blood vessels (brown, black arrow) under 5 high-power fields (200X) of normal optical microscope. (N=3) *P<.05 and ^#^*P*<.01, both versus PBS. Data shown in the graphs represent mean ± standard deviation.

To determine if downregulated miR-139-5p improves ECFC-mediated neovascularization in diabetes *in vivo*, we transplanted ECFCs and/or antagomiR-139-5p into diabetic mice after HLI surgery. Visual observations made on days 7, 14, and 21 after transplantation showed that the transplantation gradually made the skin color of the ischemic lower extremities rosy and improved the blackened appearance of the toenails. Mice treated with a mixture of ECFCs and antagomiR-139 showed the most rapid ischemic limb recovery and almost complete reversal of skin darkening and toe gangrene (Figure 10A). The mobility of the mice also significantly increased after 21 days compared with that of control mice. A significant time-dependent increase in skin temperature and blood perfusion was observed 21 days after transplantation in ECFC- (*P*<.05) and ECFC + antagomiR-139-5p-treated mice (*P*<.01) compared with PBS-treated mice (Figure 10B, 10C). Notably, the combination was more effective in reperfusing the ischemic limb than the individual agents at all time points from days 7 to 21 (Figure 10C).

Vascular density in the ischemic hind limb was assessed by CD31 expression. Vascular density was significantly increased by ECFCs (*P*<.05) and a combination of ECFC and antagomiR-139-5p (*P* < 0.01) compared with the PBS-treated group (Figure 10D).

**Figure 11:**
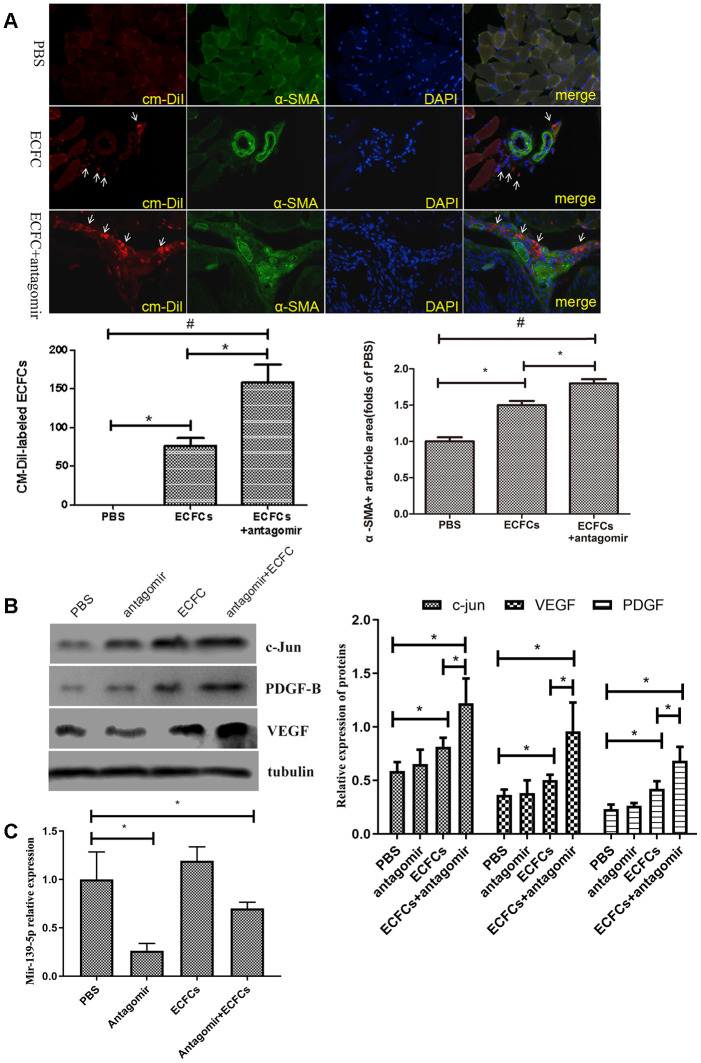
**The molecular changes in HLI diabetic mice after injecting miR-139-5p antagomir or ECFCs.** (**A**) Representative fluorescent images and quantitation of CM-DiI-labeled ECFCs (red, white arrow) and alpha smooth muscle actin (α-SMA)-positive arteriole in the ischemic adductor muscle tissue (green), as well as the merged images of CM-DiI, α-SMA, and DAPI. The representative images of arteriogenesis were expressed as α-SMA-positive arteriole area under 5 high-power fields (200X) by PBS control. (N=3) *P<.05 versus ECFCs transplantation alone group. (**B**) C-jun, PDGF-B, and VEGF expression in gastrocnemius muscle of ischemic lower extremity. (N=3) *P<.05 versus PBS or ECFCs. Data shown in all the graphs represent mean ± standard deviation. (**C**) MiR-139-5p expression in gastrocnemius muscle of ischemic lower extremity. (N=3) *P<.05 versus PBS. Data shown in the graphs represent mean ± standard deviation.

We used CM-DiI to label and trace ECFCs and further identify if the downregulation of miR-139 promotes ECFCs incorporation into the ischemic tissue endothelium. CM-DiI-labeled ECFCs were seen in the gastrocnemius muscle of the lower extremity and around the vessels on day 14 after transplantation (Figure 11A). Compared with the ECFCs group, CM-DiI labeled cells in the ECFC + antagomiR-139-5p combination group were higher than in the ECFCs group (*P*<.05), and α-SMA + arteries in the antagomiR-139-5p combination group also increased (Figure 11A). These results suggest that ECFCs could be a potential player in angiogenesis, and that miR-139 downregulation could maintain the survival of ECFCs, and thus, promote angiogenesis.

We used Western blot to assess whether the VEGF, PDGF-B, and c-jun signaling pathway proteins are involved in miR-139-mediated angiogenesis in ECFCs. It was found that ECFC + antagomiR-139-5p combination and ECFCs alone significantly increased VEGF, PDGF-B, and c-jun expression in the gastrocnemius muscle of the ischemic lower extremities, and there were differences between the two groups (*P*<.05; Figure 11B). Thus, downregulation of miR-139-5p expression in ECFCs promotes activation of c-jun, VEGF, and PDGF-B signaling (Figure 11C), indicating that miR-139-5p is required for c-jun activation in diabetes, which is at least partly related to a reduction in VEGF and PDGF-B response in diabetic ECFCs.

## DISCUSSION

The EPCs from diabetic patients present decreased number and impaired angiogenic function, which plays a causative role in the development and progression of virtually all diabetic complications [32]. Despite its critical importance, the mechanism for impaired ECs function in diabetes remains largely unknown. The present study provides two new lines of evidence implicating miR-139-5p/c-jun-initiated pathways in diabetic ECs dysfunction: 1) The existence of strong relationship between miR-139-5p levels and diabetic ECs dysfunction, since diabetic ECFCs and ECs display higher miR-139-5p levels, and diabetic dysfunction could be mimicked by upregulation of miR-139-5p levels and reversed by miR-139-5p knockdown. 2) The benefit of decreased miR-139-5p expression occurs through the activation of c-jun signaling and VEGF and PDGF-B expression induction in diabetic ECs.

It is known that miR-139-5p inhibits cell proliferation and angiogenesis in many types of cancer [16–21]. Li et al. demonstrated that miR-139-5p expression was increased in the pancreas of diabetic rats, and downregulation of miR-139-5p contributed to the anti-apoptotic effect on the diabetic rat pancreas and INS-1 cells [22]. In the present study, we demonstrated that miR-139-5p expression significantly increased in ECFCs of diabetic patients and ECs of diabetic rats and diabetics nude mice. Furthermore, normal ECFCs functions such as proliferation, migration, and tube formation were attenuated after transfection with miR-139-5p mimics, while those functions were rescued in diabetic ECFCs transfected with miR-139-5p inhibitors. This indicates that miR-139-5p is a strong candidate for impairment of ECs function under diabetic conditions.

C-jun/AP-1 has been implicated in complications such as diabetic embryopathy [33] and retinopathy [34]. It is also involved in feedback inhibition of insulin release by phosphorylating the rat/mouse insulin receptor substrate 1 [35]. Apart from being involved in the negative regulation of insulin release, c-jun activity contributes to vascular insulin resistance and ECs dysfunction [36]. In the present study, we observed that c-jun expression was strongly inhibited by miR-139-5p mimics in healthy subjects-derived ECFCs, whereas c-jun expression was resumed by miR-139-5p inhibitors in diabetic-derived ECFCs. Consistently, c-jun siRNA significantly attenuated miR-139-5p deprivation-induced tube formation and cell migration of ECFCs. Recently, it has been reported that miR-139-5p is down-regulated in the heart of hypertrophic cardiomyopathy patients, and it inhibits cardiac hypertrophy by targeting expression of c-jun [37]. Zhang et al. [17] demonstrated that miR-139 could inhibit c-jun expression by targeting a conserved site on its 3′-UTR, with miR-139 downregulation being inversely correlated with c-jun expression in human gastric cancer. Restoration of miR-139-5p expression in gastric cancer cells induced apoptosis and inhibited cell migration and proliferation by targeting Jun. On the basis of these researches and our own results about miR-139-5p and c-jun 3′-UTR binding, we hypothesized and finally confirmed that miR-139-5p blocked the function of ECs via the c-jun pathway. It is well known that miRNAs usually display regulation function through multiple target genes. Although our research demonstrated that upregulation or downregulation of miR-139-5p did not change the expression of IGF1R, other target genes still might be involved. Nevertheless, our results show that c-jun is an important target gene that mediates the regulation of miR-139-5p on diabetic endothelial cells.

C-jun is a major component of the AP-1 transcription factor, which is composed of a mixture of homo- and heterodimers formed by proteins c-jun and c-fos, and binds to the promoters of various genes to regulate their expression. The expression of VEGF—an important factor in neovascularization—is promoted by factors such as HIF-1α [38] and AP-1, which is activated by the AKT/mTOR (mammalian Target of Rapamycin) pathway [39]. Yin et al. [29] demonstrated that phosphorylated c-jun alone could recognize and bind to the cis-elements of the VEGF promoter and then activate VEGF transcription. On the basis of these studies, we hypothesized that miR-139-5p could inhibit the expression of VEGF via the c-jun pathway in ECs. We found that transfected healthy subjects-derived ECFCs with c-jun plasmid rescued miR-139-5p mimic-induced decrease in VEGF expression. In contrast, transfected diabetic-derived ECFCs with c-jun siRNA blocked VEGF induction by miR-139-5p inhibitors. Furthermore, ChIP experiments show miR-139-5p mimics significantly decreased c-jun-VEGF promoter activity. Thus, we proved that miR-139-5p can inhibit VEGF expression in ECFCs partially via c-jun and by reduction in c-jun-VEGF promoter activity. Since VEGF induces EPCs proliferation and angiogenic function mainly through receptor tyrosine kinase KDR and Flk-1, and VEGF-KDR signaling pathway as positively feedback mechanism induces KDR expression in endothelial cells [40], therefore, pretreatment of ECFCs with anti KDR further demonstrated that VEGF is an important intermediate mediating the effects of miR-139-5p on ECFCs.

PDGF is highly homologous with VEGF, which is also a critical angiogenic factor. PDGF is required for the recruitment and differentiation of various cells including pericytes and smooth muscle cells in the maturation of blood vessels [41, 42]. It is known that PDGF-B can be secreted by the ECs during angiogenesis, which facilitates recruitment and proliferation of perivascular cells [4]. It was also found that PDGF-B secreted by EPCs increases ECs and EPCs migration and proliferation as well as tubule structure formation by PDGFR-B signaling axis [43, 44]. Similar to VEGF, the expression of PDGF-B was also regulated by the AP-1 transcription factor. The stimulatory effect of PDGF on transin transcription involved factors recognizing the sequence TGAGTCA, which is found in the transin promoter and is reported to be a binding site for the transcription factor Jun/AP-1 [45]. Based on these reports, we explored whether miR-139-5p could decrease the PDGF expression through c-jun/AP-1. In the present study, we have clearly proved that diabetic ECFCs with a high level of miR-139-5p expressed less PDGF-B, which could be reversed by transfecting c-jun plasmid. Anti-PDGF-B treatment in ECFCs significantly decreased angiogenesis induced by miR-139-5p inhibitor. These results indicate that miR-139-5p could also inhibit angiogenesis by reducing PDGF expression mediated by c-jun pathway.

EPCs promote vascular repair and homeostasis and may be used as a potential biomarker for atherosclerosis and vascular disease [46]. ECFCs is a key subgroup of EPCs involved in angiogenic repair properties. Decreased number and impaired function of ECFCs derived from diabetic patients were found. Therefore, it is of interest to verify if miR-139-5p downregulation could reverse the defects in diabetic-derived ECFCs. Although preclinical models such as HLI are commonly used to assess angiogenesis, the establishment of such models in the absence of cardiovascular risk factors (e.g., diabetes) hampers the observed results’ clinical translation [47]. Hence, we used diabetic mice to establish HLI. Similar to the *in vitro* results, miR-139-5p downregulation rescued ECFC-mediated neovascularization in the ischemic limb through the c-jun/VEGF and PDGF pathway. Moreover, ECFCs were also incorporated in the ischemic tissue endothelium. These results showed the congruence between *in vitro* and *in vivo* results.

Clinically, multiple studies have focused on the therapeutic effect of mesenchymal stem cells (MSCs)/EPCs in diabetes or its complications. Kirana et al. [48] reported that using bone marrow-derived CD90^+^ stem cells for diabetic foot ulcer treatment improved the microcirculation and supported wound healing. Das et al. [49] observed significant pain relief, ulcer healing, and blood flow restoration in patients with critical limb ischemia (CLI) after intra-arterial allogenic MSCs transplantation. Adipose-derived MSCs have been reported to improve the formation of numerous collateral vessels after multiple intramuscular transplants in patients with CLI [50]. Adoption of an MSCs-EPCs combination for the treatment of CLI patients was also considered to be efficacious as the walking function and blood perfusion were largely improved [51]. Apart from treating ischemia, MSCs have also been deemed successful in increasing insulin sensitivity [52] as well as offering long-term hyperglycemia control [53]. Therefore, promoting MSCs/EPCs therapy may contribute to improvements in diabetic patient outcomes, and miR-139-5p/c-jun may also be a target to improve the treatment of EPCs.

## CONCLUSIONS

In conclusion, high miR-139-5p expression due to diabetic conditions inhibits the c-jun pathway and downregulates VEGF and PDGF-B expression, leading to a decrease in ECFC migration, proliferation, and tube formation, which subsequently reduces EC survival. Therefore, diabetes-induced miR-139-5p upregulation might be an important therapeutic target in the repairment and regeneration of diabetic vascular injury.

## MATERIALS AND METHODS

### Isolation and culture of ECFCs and ECs

The study was approved by the Third Xiangya Hospital Review Board. Four healthy volunteers [age: 43.3±14.9 (range 39-59) years, BMI: 23.58 ± 3.93 kg/m^2^] and four patients with type 2 diabetes [age: 46.5 ± 9.0 (range 28-56) years, BMI: 24.64 ± 6.19 kg/m^2^] were recruited from the Third Xiangya Hospital. The diagnose of type 2 diabetes was based on the 2010 guidelines from the American Diabetes Association [54]. All participants had no severe organ dysfunction and the diabetes duration of the type 2 diabetic patients was no more than 5 years. Human ECFCs were isolated as described previously [28]. Briefly, peripheral blood (PB) samples (20 mL) were collected in heparinized solution. Human mononuclear cells that were centrifuged, isolated, and re-suspended in complete EGM-2 medium (Lonza, Rockland, ME, USA) were seeded to 6-well tissue culture plates (5 × 10^7^ cells/well) precoated with type 1 rat tail collagen (BD Biosciences, Bedford, MA, USA) and cultured in a humidified incubator. After culturing for 24 hours, adherent cells were washed with complete endothelial cell growth medium 2 (EGM-2), and the same medium was added to each well. The medium was changed daily for a week and then every other day until the first passage.

ECFCs appeared within 12 to 16 days of culture and were identified as circumscribed monolayers of cobblestone-appearing cells. Upon confluence, the ECFCs were detached with 0.25% trypsin-ethylenediaminetetraacetic acid (EDTA; Invitrogen, Carlsbad, CA, USA), resuspended in complete EGM-2 media and plated on tissue culture flasks coated with type 1 rat tail collagen. Isolated ECFCs were cultured to passages 3-5 for cell assays and transplantation.

ECs were isolated from diabetic rat and diabetic nude mouse aorta as described previously [55]. Briefly, the aorta of the diabetic animal was dissected out from the aortic arch to the abdominal aorta and immersed in 20% fetal bovine serum Dulbecco’s modified Eagle’s medium (FBS-DMEM) containing 1000 U/mL of heparin. After fat or connective tissue removal and ligation, the aorta was filled with collagenase type II solution and incubated at 37° C for 45 minutes. ECs were removed from the aorta by flushing with 5 mL of DMEM (Gibco, Life Technologies, Shanghai, China) containing 20% FBS (Gibco, Life Technologies, Mulgrave, VIC, Australia) and collected by centrifugation at 1200 rpm for 5 minutes. The precipitate was then re-suspended by a pipette with 2 mL of 20% FBS-DMEM and cultured in a 35-mm collagen type 1-coated dish. After incubating for 2 hours at 37° C, the medium was removed, the cells were washed with warmed phosphate-buffered saline (PBS), and the medium was added. One week later, confluent ECs were harvested for assays.

Human umbilical vein endothelial cells (HUVECs) were purchased from the cell biology research laboratory of Central South University (Changsha, Hunan Province, China).

### Cell transfection

MiR-139 mimic (miR10000250), mimic-NC, inhibitor (miR20000250), inhibitor-NC, antagomiR-139 (miR30000250), and antagomiR-NC (NC: negative control) were synthesized by RiboBio (RiboBio, Guangzhou, China). C-jun siRNA were purchased from GenePharma (Shanghai, China), and c-jun plasmids were purchased from Vigene (Jinan, China). The final concentration of miR-139 mimics, miR-139 inhibitor and c-jun siRNA in the transfection system was 100nM, respectively. The concentration of c-jun plasmid in the transfection system was 1ug/ml. When the confluence of ECFCs was approximately 70% to 80%, the miR-139 mimic, miR-139 inhibitor, c-jun siRNA, and c-jun plasmids were transfected with Lipofectamine 3000 (Invitrogen, Carlsbad, CA, USA). After 72 hours, the cells were subjected to the process of induction of differentiation.

### Cellular assays

Isolated ECFCs were cultured to passages 5-7 for cellular assays. Tube formation assays were performed as described previously [56]. Briefly, ECFCs or HUVECs were seeded onto 96-well tissue culture plates coated with 30μL Matrigel (BD Biosciences, San Jose, CA, USA) at a cell density of 5x10^3^ to 2x10^4^ cells/well. Cells were observed every 2 hours by visual microscopy with an inverted microscope (Olympus, Lake Success, NY, USA) at 40X magnification for capillary-like formation. Colony-forming assays were performed as described previously [28] using limiting dilution assays. ECFCs were seeded in 6-well plates precoated with type 1 rat tail collagen (200 cells/well) with each condition plated in triplicate. On day 14 after initial plating, wells were scored for colony formation by visual inspection with an inverted microscope under 40X magnification. CCK-8 assays were performed for evaluation of cell proliferation. Briefly, cells were seeded in 96-well plates at a density of 10^4^ cells/well and incubated at 37° C for 24 hours. Then 10 μL of CCK-8 solution (Sangon Biotech, Shanghai, China) was added to each well of the plate. After incubation for 1 to 4 hours, the absorbance at 450 nm was measured.

### Bioinformatic analysis

MiR-139-5p expression profiling data were downloaded from the NCBI GEO database. TargetScan 6.2 (http://www.targetscan.org), PicTar (http://pictar.mdc-berlin.de), and PITA (genie.weizmann.ac.il/pubs/mir07/mir07_prediction.html) were used to predict miR-139 targets. These targets were predicted and downloaded from the respective databases with default or stringent settings and then compared and combined with the NCBI GEO database. Only targets that were confirmed by at least 2 methods were selected for further analyses.

### Luciferase reporter assay

Luciferase reporter assays were performed as described previously [57]. Briefly, the reporter plasmids were co-transfected using Lipofectamine 2000 reagent (Invitrogen, Carlsbad, CA, USA) with the miR-139-5p mimics and the vector phRG-TK (Promega, Madison, WI, USA), which expresses synthetic Renilla luciferase to normalize the transfection efficiency. Luciferase activities were measured using the Dual-Luciferase Reporter Assay reagent (Promega, Madison, WI, USA) on the LB 960 Centro XS3 luminometer (Berthold Technologies, GmbH & Co. KG, Bad Wildbad, Germany).

### Real-time reverse transcription polymerase chain reaction (PCR) for miRNA and mRNA analyses

The total RNA was isolated with TRIzol reagent (Life technology) and reverse transcription performed using oligo dT primers or random N6 primers for mRNA and stem-loop RT primer for miRNA with Superior III RT Supermix (Innogene biotech, Beijing, China). The real-time PCR primers used are available on request. Real-time PCR was performed using the Eppendorf realplex with UltraSYBR Mixture (Toyobo Co., Osaka, Japan), the expression was quantified relative to the housekeeping gene (glyceraldehyde-3-phosphate dehydrogenase) for mRNA and U6 for miRNA.

### Western blot assay

Protein homogenates were separated on a 12% sodium dodecyl sulfate (SDS)-polyacrylamide gel electrophoresis with a constant voltage of 75 V. And then electrophoresed proteins were transferred to nitrocellulose membranes with a transfer apparatus (Bio-Rad). The membranes were blocked with 5% non-fat milk in Tris-buffered saline (pH 7.5) with 0.05% Tween 20 for 2 hours at room temperature. Primary antibodies including cJun (Cell Signaling Technology, Danvers, MA, USA), VEGF (Cell Signaling Technology, Danvers, MA, USA), PDGF-B (#ab23914, Abcam, Cambridge, USA), and β-Actin (#A5316, Sigma-Aldrich, St. Louis, MO, USA) were diluted in antibody binding buffer overnight at 4° C. The blots were washed 3 times in TBS buffer for 10 min and then immersed in the second antibody solution containing goat- IgG mouse polyclonal antibody, goat- IgG mouse polyclonal antibody (Proteintech Chicago, USA) for 2 h and diluted in TBS buffer. The membranes were washed 3 times for 10 min in TBS buffer. The immunoblotted proteins were visualized using an ECL Western blotting luminal reagent (Advansta, USA) and quantified using a universal Hood II chemiluminescence detection system (Bio-Rad, USA).

### ChIP-PCR

ChIP was performed using the EZ-Magna ChIP™ G-Chromatin immuno-precipitation kit (Millipore, 17-409, Darmstadt, Germany) following the manufacturer’s guide with c-Jun antibody (#24909-1-AP, Proteintech, Wuhan, China) or IgG control (#2729, Cell Signaling Technology, Danvers, MA, USA). Fresh culture medium containing 1% formaldehyde was added to the culture and kept for 10 minutes at room temperature and then lysed with ChIP lysis buffer for 10 minutes at room temperature to isolate the nuclear pellets. Chromatin solutions were sonicated for 4 pulses of 12 seconds each at 70W using a sonic probe, followed by a 40-second rest on ice between each pulse to generate 200 to 1000 bp DNA fragments. Supernatants were collected and 5μL was removed as “input DNA.” Chromatin was then subjected to immunoprecipitation for 1 hour at room temperature using strip wells precoated with antibodies specific to c-jun or normal mouse IgG. After 6 washes with the buffer, the immunoprecipitated chromatins and input DNA coated on the strip wells were digested with proteinase K and purified using collection tubes. Purified DNA was subjected to Plasmid-Safe ATP-Dependent DNase digestion and real-time PCR amplification, as described above.

### KDR and PDGF-B antibody pretreatment

In order to examine the effects of miR-139-5p inhibitor on the biological functions of ECFCs that depend on PDGF-B and VEGF-A, the groups of ECFCs were treated with KDR antibody (89106, 1:100, BD Biosciences, Bedford, MA, USA) and PDGF-B antibody (743035, 1:100, BD Biosciences, Bedford, MA, USA), respectively, or both, and incubated with or without transfected miR-139-5p inhibitor for 48 hours; then the cells were digested by trypsin and seeded on Matrigel for tube formation assays.

### Matrigel plug assays *in vivo*

Matrigel plug assays *in vivo* were performed as described previously [58]. Matrigel (500 μL) containing normal/diabetic ECFCs (2 × 10^6^ cells/gel) transfected with miR-139-5p mimics/inhibitors or PBS was injected subcutaneously into the abdomen of nude mice (n = 4). Two weeks later, the Matrigel was removed and prepared for paraffin sections. Post-preparation, the blood vessels were stained with CD31 and examined by microscope (Olympus, Lake Success, NY, USA) under 200X magnification.

### Establishment of a diabetic model in rats and nude mice

Diabetic model in rats and nude mice was used to evaluate changes in miR-139-5p expression in aortic endothelial cells, whereas mice were used for hind limb ischemia (HLI) model establishment. Nude mice (SPF grade) and Sprague-Dawley rats (both purchased from Hunan SJA Laboratory Animal Co., Ltd, Hunan, China) were used in this study. The animals were maintained under specific conditions in accordance with the regulations of the Instructional Opinions on Ethical Treatment of Animals. The study was approved by the Animal Ethics Committee of Xiangya Third Hospital of Central South University. 8-week-old male Sprague-Dawley rats in the control group (n = 6) and disease group (n = 6) were fed with normal forage or high fat and sugar forage, respectively, for 4 weeks. In the fifth week, the disease group was injected with streptozotocin (STZ, 40 mg/kg body weight; Sigma-Aldrich, St. Louis, MO, USA) intraperitoneally to establish diabetes and observe changes in miR-139-5p expression in endothelial cells of the thoracic aorta.

STZ (150 mg/kg body weight) was also administered intraperitoneally to forty 10-week-old weighed male nude mice after fasting for 12 hours. Blood glucose levels were measured on days 3 and 7 after STZ injection. The mice with blood glucose level higher than 16.7 mmol/L and symptoms of polyuria and polydipsia were considered as having diabetes. Mice with blood glucose level <16.7 mmol/L were excluded from the study. Diabetes was successfully induced in 38 (88.3%) mice. Ten 10-week-old male nude mice raised with normal forage were used as control group.

### Hind limb ischemia model and limb perfusion ratio assessment

Diabetic nude mice were used to develop the HLI model as described previously [59]. After anesthetizing with 1% pentobarbital intraperitoneal injection, the entire right superficial femoral artery and vein (from just below the deep femoral arteries to the popliteal artery and vein) were ligated with 8-0 silk sutures, cut and excised with an electrical coagulator. The overlying skin was closed with 5-0 silk sutures. The mice were returned to the cage after awakening with no restriction on feeding.

After surgery, 30 mice were selected and randomly divided into 5 groups. Mice in group A received sham surgery, group B received intramuscular injection of PBS, group C received intramuscular injection of antagomir-139-5p, group D received intramuscular injection of 1 × 10^6^ CM-DiI-labeled (CellTracker^TM^ C7000, Invitrogen, Carlsbad, CA, USA) ECFCs, and mice in group E were injected with mixture of antagomir-139-5p and CM-DiI-labeled ECFC.

In order to evaluate the limb perfusion ratio (ischemic limb [right]/normal limb [left]), a real-time microcirculation imaging analysis was performed using a PeriCam Perfusion Speckle Imager (PSI) based on the laser speckle contrast analysis technology (Perimed Inc., Kings Park, NY, USA) at days 0, 7, 14, and 21 after ischemia. Meanwhile, skin temperature was measured using a digital thermometer.

### Histological assessment and detection of vascular density

After antigen retrieval and endogenous peroxidase blockade, the dewaxed paraffin section of muscle tissue was incubated overnight with rabbit anti-mouse CD31 polyclonal antibody (1:100, Proteintech, Chicago, IL, USA) at 4° C. The slide was washed and incubated with 150μL mouse anti-rabbit secondary antibody (1:200) at room temperature for 2 hours. The slide was then subjected to blockage with biotin and avidin (ABC) solution, color development with 3,3′-diaminobenzidine (DAB) solution, dehydration, and sealing. Under normal optical microscope, 5 high-power fields (200X) were randomly selected for each slice to count the number of vWF-stained blood vessels (brown) as vascular density. One single stained vascular endothelial cell or vascular endothelial cell cluster was considered as a vascular count. Blood vessels containing more than 8 red blood cells within the tube or displaying a thick muscle layer were excluded as vascular count. Morphological changes of muscle tissue were observed by hematoxylin staining under normal optical microscope.

### ECFCs tracing

In order to examine the homing and incorporation of ECFCs in the ischemic muscle, ischemic gastrocnemius muscle and/or soleus muscle were collected on day 14 and embedded in optimal cutting temperature medium for the frozen section. Fluorescence microscope was utilized to find CM-DiI-labeled cells (red-colored) on the section to track migration and differentiation of ECFCs after transplantation. The CM-DiI-labeled ECFCs were counted in randomly selected fields for a total of 20 different fields (40X magnification) per section and 3 sections per animal. Frozen sections were stained with anti-alpha smooth muscle actin (α-SMA) antibody (Abcam, Cambridge, MA, USA) to evaluate the arteriole area. The arteriole area was calculated in randomly selected fields for a total of 20 different fields (40X magnification) per section and 3 sections per animal. The arteriole area was expressed as α-SMA-positive arteriole area per fiber and normalized by control.

### Statistical analysis

All data are presented as mean ± standard deviation. Two groups were compared by the unpaired Student’s t-test and multiple groups were analyzed by 1-way analysis of variance (GraphPad Prism, La Jolla, CA, USA). The statistical significance was defined as *P*<.05.

## Supplementary Material

Supplementary Figures
