# Supplementary material for: MicroRNA-139-5p upregulation is associated with diabetic endothelial cell dysfunction by targeting c-jun
Source: Aging (Albany NY). 2020 Dec 3;13(1):1186–211. doi: 10.18632/aging.202257 (PMC7835005; doi:10.18632/aging.202257)
Supplement: Supplementary Figures [file aging-13-202257-s001.pdf]

SUPPLEMENTARY FIGURES

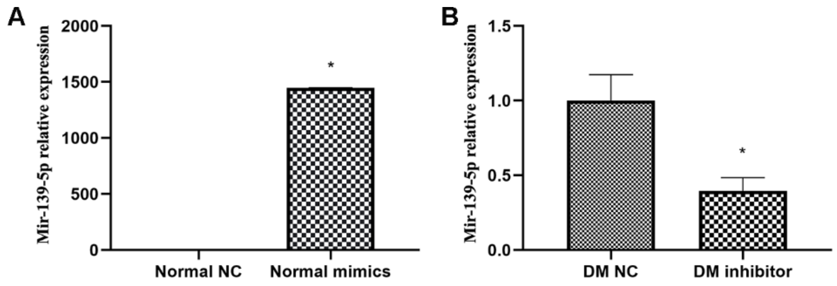

Supplementary Figure 1. MiR-139-5p expression in ECFCs from healthy or diabetic subjects when treated with miR-139-5p mimics or inhibitors. MiR-139-5p expression was detected by real-time PCR in healthy adults derived-ECFCs transfected with miR-139-5p mimics (**A**) and in diabetic patients-derived ECFCs transfected with miR-139-5p inhibitors (**B**). (N=3) *P<.05 versus control. Data shown in the graphs represent mean ± standard deviation.

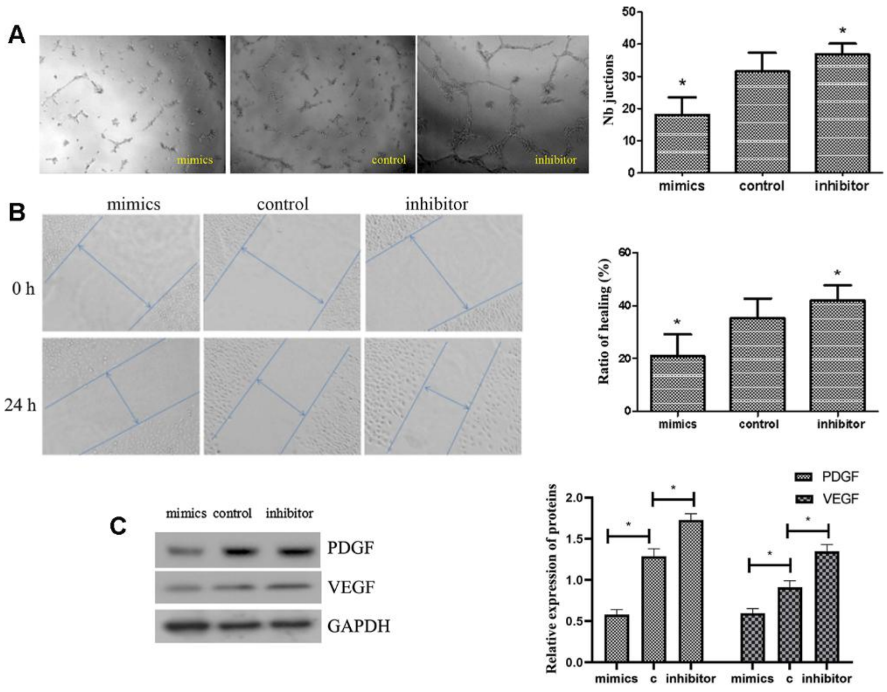

Supplementary Figure 2. (**A**, **B**) Tube formation and migration of HUVECs transfected with miR-139-5p mimics or inhibitors. The photos were captured by a 40X microscope and statistics performed by Image J. (N=3) *P<.05 versus control. (**C**) PDGF-B and VEGF expression in HUVECs transfected with miR-139-5p mimics or inhibitors. (N=3) *P<.05 versus control. Data shown in the graphs represent mean ± standard deviation.

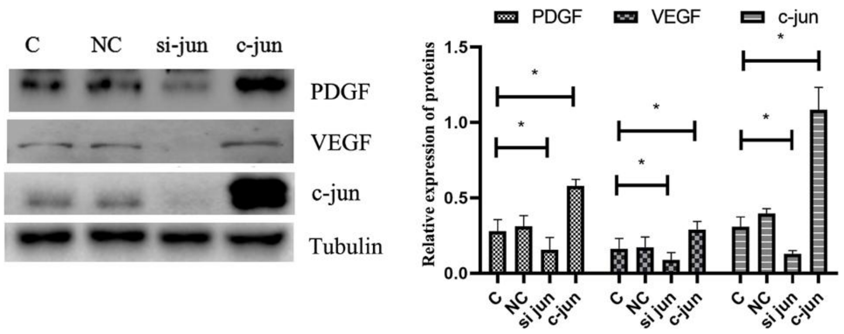

Supplementary Figure 3. C-jun, PDGF-B, and VEGF expression after transfection with c-jun siRNA or c-jun plasmid in diabetic ECFCs. PDGF-B and VEGF expression of normal ECFCs transfected with si-jun and DM ECFCs transfected with c-jun plasmid was detected by Western blot. (N=3) **P*<.05 versus diabetic ECFCs. Data shown in the graphs represent mean ± standard deviation.
